# A Gated Recurrent Unit based architecture for recognizing ontology concepts from biological literature

**DOI:** 10.1186/s13040-022-00310-0

**Published:** 2022-09-28

**Authors:** Pratik Devkota, Somya D. Mohanty, Prashanti Manda

**Affiliations:** 1 Department of Computer Science, University of North Carolina at Greensboro, Greensboro, USA; 2 Informatics and Analytics, University of North Carolina at Greensboro, Greensboro, USA

**Keywords:** Deep learning, Gene ontology, Automated annotation, Scientific literature

## Abstract

**Background:**

Annotating scientific literature with ontology concepts is a critical task in biology and several other domains for knowledge discovery. Ontology based annotations can power large-scale comparative analyses in a wide range of applications ranging from evolutionary phenotypes to rare human diseases to the study of protein functions. Computational methods that can tag scientific text with ontology terms have included lexical/syntactic methods, traditional machine learning, and most recently, deep learning.

**Results:**

Here, we present state of the art deep learning architectures based on Gated Recurrent Units for annotating text with ontology concepts. We use the Colorado Richly Annotated Full Text Corpus (CRAFT) as a gold standard for training and testing. We explore a number of additional information sources including NCBI’s BioThesauraus and Unified Medical Language System (UMLS) to augment information from CRAFT for increasing prediction accuracy. Our best model results in a 0.84 F1 and semantic similarity.

**Conclusion:**

The results shown here underscore the impact for using deep learning architectures for automatically recognizing ontology concepts from literature. The augmentation of the models with biological information beyond that present in the gold standard corpus shows a distinct improvement in prediction accuracy.

## Background

Ontologies have become the de-facto mode of representing biological knowledge since the development of the Gene Ontology (GO) [1]. Following the widespread adoption of the GO, other bio-ontologies representing knowledge in disparate aspects of biology and biomedicine have been created. Today, an estimated 958 bio-ontologies are in use spanning over 55 million annotations (as of 1-20-22 from https://bioportal.bioontology.org/). While the use of bio-ontologies and the number of annotations created using these ontologies have grown exponentially, the methods used to create these annotations haven’t changed at a comparable pace. The majority of ontology annotations are still created via manual curation - the process where a human curator reads scientific literature and manually selects appropriate ontology concepts to describe phrases/words in the text. The process of manual creation is slow, tedious, and unscalable to the rapid pace of scientific publishing [2].

Over the past decade, text mining approaches have been developed to conduct ontology annotation in an automated manner. Preliminary solutions include syntactic, lexical approaches followed by traditional machine learning applications [3]. Lexical solutions for automated ontology annotation rely on similarities between a piece of text and the name of an ontology concept or their synonym to assign annotations [4]. This approach can be challenging when the text does not match the names of ontology concepts. Also, some ontology concept names contain a large number of words which makes text matching difficult [4].

Text mining tools that use machine learning based methods employed supervised learning techniques using gold standard corpora [3]. These methods can form generalizable associations between text and ontology concepts leading to improved accuracy. The rise of deep learning in the areas of image and speech recognition has translated into text-based problems as well. Preliminary research has shown that deep learning methods result in greater accuracy for text-based tasks including identifying ontology concepts in text [5–9]. Deep learning methods use vector representations that enable them to capture dependencies and relationships between words using enriched representations of character and word embeddings from training data [10].

The semantic complexities of identifying the appropriate ontology concept for a word/phrase are quite challenging. In the simplest case, the name of the ontology concept is an exact match to the piece of text. For example, the phrase “brain development” in the sentence “HOMER proteins have also been implicated in axon guidance during brain development” is annotated to the GO term “brain development (GO:0007420)”. Sometimes, a match can also be made by comparing the text to the names of known synonyms of concepts in the ontology. In most cases, there aren’t clear matches between the words being annotated to the names of the ontology concepts. For example, the word “olfactory” in the sentence “Class I olfactory receptors are bracketed, and the remaining olfactory receptors are class II.” is annotated to the GO term “sensory perception of smell (GO:0007608)”. 80% of the annotations made in the latest version of the CRAFT corpus have no clear match between the text and the name of the ontology concept used for annotation. This is a clear indication of the complexity of the problem at hand, one that cannot be solved just by syntactic methods or by text matching. These are the cases where effective training can make a substantial difference.

Training deep learning models requires good quality training datasets. The Colorado Richly Annotated Full Text Corpus (CRAFT) [11] is a widely used training resource for automated annotation approaches. The current version of the CRAFT corpus (v4.0.1) provides annotations for 97 biological/biomedical articles with concepts from 9 ontologies including the GO. CRAFT uses a number of formats with different levels of complexity to represent annotations.

One of the challenges in creating effective deep learning models is translating all of CRAFT’s annotations to formats that can be leveraged by the models. This process involves a substantial amount of preprocessing that’s designed specifically for each annotation format to ensure that each annotation is represented soundly and completely in the training data. Another challenge when using machine learning solutions - including deep learning models is the availability and abundance of training data. Not all concepts in the ontology are represented in the gold standard corpus hindering the ability of the trained models to recognize those unseen concepts. Among the concepts that are present in the training data, some of them occur frequently while others are sparse. It might be necessary to augment the primary training corpus with information from other sources to improve prediction accuracy.

The choice of deep learning model and architecture also impacts prediction performance. We have conducted comparisons of models such as CNNs, GRUs, LSTMs, RNNs in previous work [9, 12] whose findings enable us to making informed choices in this study. Here, we present a deep learning architecture that leverages inputs from multiple sources and in different formats (characters, words, etc.) to improve on the state-of-art in terms of prediction performance.

We make two contributions in this study - 1) publicly available preprocessed annotations from the CRAFT corpus for training deep learning models and 2) deep learning architectures for identifying ontology concepts.

### Related work

Substantial work has been conducted in the area of employing automated methods for identifying ontology annotations. The majority of this work is geared towards identifying GO annotations since the GO is the most widely used biological ontology. Some of the preliminary work in this space was aimed to assign GO terms to protein sequences and not to free text in literature.

Similarity based approaches identify GO annotations based on similarity between protein sequences [13–15]. When a sequence database is searched for a protein sequence, GO terms associated with similar sequences retrieved from the search are assigned to the query sequence. Probabilistic methods assume that the probability of shared GO functions is higher between proteins in close proximity on a protein interaction graph [16–20]. Markov Random Fields and Bayesian frameworks were used to determine probability of shared GO functions in these approaches. Later, machine learning approaches such as Support Vector Machines were used to identify hidden relationships between protein features such as sequences, structure, etc. to annotate new proteins [21–24]. The latest developments in this area employ deep learning models for the task of automatically annotating proteins with GO terms. Various supervised deep learning architectures like Long Short Term Memory (LSTM), Convolutional Neural Networks (CNN), Recurrent Neural Networks (RNN), Gated Recurrent Units (GRU), and Bidirectional RNNs have been shown to perform well at this task.

The early use of automated concept annotation had set the stage for more sophisticated problems such as associating ontology concepts to pieces of text from scientific literature. The task of automatically annotating scientific literature with ontology concepts is the task of focus in our study. Preliminary studies in this area employed the use of lexical, syntactical, and traditional machine learning [3]. In prior work, we presented a review of these approaches and conducted a performance comparison using a gold standard dataset [3]. However, in more recent years, the state of art has evolved to leverage deep learning models due to the promise of increased accuracy and speed [5–8]. In addition, deep learning models can develop richer representations of the input training data by using vector representations that capture dependencies between words, characters, and sequence structures. In the next section, we will discuss applications that use deep learning for automated ontology annotation of text.

In early uses of deep learning for ontology annotation of text, CNNs combined with LSTMs were used [25]. The work provided a proof-of-concept for the use of deep learning for ontology annotation and showed improved performance over traditional, machine learning methods. Other studies conducted performance comparisons among deep learning models and found that CNNs with enhanced inputs such as character embeddings were particularly effective for biomedical named entity recognition [26].

In a previous study [12], we presented a deep learning architecture that used multiple GRUs with a character+word based input. The model was compared to seven models from existing work using the CRAFT corpus as a gold standard. Results showed that our GRU-based model outperformed prior models. This work was limited to predicting unigram annotations and did not take into account the rich semantic information in ontology hierarchies. Subsequent work [9] from our group improved on this by expanding the types of annotations predicted and by incorporating semantics from ontology subsumption into the prediction. Surprisingly, we found that GRU based models consistently outperformed the commonly used LSTM based architectures. Contrary to expectations, the inclusion of ontology hierarchy resulted in a modest improvement in performance [9].

Most recent publications in this area have separated the ontology annotation task to two sub-tasks - 1) span detection: detecting the part of text that corresponds to an ontology concept, and 2) concept normalization: identifying the ontology concept most appropriate for the identified piece of text [27, 28]. Using the CRAFT corpus as a training set, the study reports that Bidirectional encoder representations from transformers for biomedical text mining (BioBERT) resulted in the best performance (0.81 F1) for the span detection sub-task. The Open-source toolkit for Neural Machine Translation (OpenNMT) yielded the best performance for concept normalization. Overall, their results suggest that their approach using BioBERT for span detection and OpenNMT for concept normalization achieved state-of-the-art performance for most ontologies in CRAFT corpus while using substantially fewer computational resources.

Treating the ontology annotation task as a sequence-to-sequence problem, another study [29] compared the performance of an LSTM model with BERT. This study divided the ontology annotation task into span detection and named entity normalization (NEN). However, instead of treating the steps like a pipeline where the output for the first step feeds into the next, these steps are carried out independently and agreement between the predictions is examined. The work uses ontology pretraining using names and synonyms of concepts found in the ontology. This step enables the models to predict concepts that might not be seen in the training data. The pretraining is further combined with a rule-based dictionary-lookup system that directly queries concept names from the ontology. Results show that the pretraining and lookup systems improve performance. The study reports an F1 score of 0.84 using a bidirectional LSTM based architecture. Note that this system currently cannot handle sophisticated annotation formats such as discontinuous and overlapping annotations as represented in the CRAFT corpus.

## Methods

### Training Dataset

This study used version v4.0.1 (https://github.com/UCDenver-ccp/CRAFT/releases/tag/v4.0.1) of The Colorado Richly Annotated Full Text Corpus (CRAFT) [11], a manually annotated corpus containing 97 articles each of which is annotated to 9 ontologies. All of the articles in the CRAFT corpus are part of the PubMed Central Open Access Subset. We selected GO annotations from the CRAFT corpus as our training and testing set because the largest number of annotations in CRAFT are made using the GO.

### Data Preprocessing

Each of the 97 articles in the CRAFT corpus has a corresponding xml annotation file which describes annotations within the sentences using character indexes of the article. The first step is to preprocess each annotation into a format that can be used by the deep learning models. All 97 articles are read as UTF-8 encoded strings and the corresponding xml file for each article is parsed. The following preprocessing steps are performed to translate annotations from the CRAFT corpus to the desired input formats for the deep learning models.

#### Sentence segmentation and Tokenization

As mentioned earlier, annotations for each CRAFT article are recorded in the corresponding xml annotations file via character index spans. The following is an example of a sentence and its corresponding annotation:

**Sentence:** *“We observed a severe autosomal recessive movement disorder in mice used within our laboratory.”*

**Annotation:** 
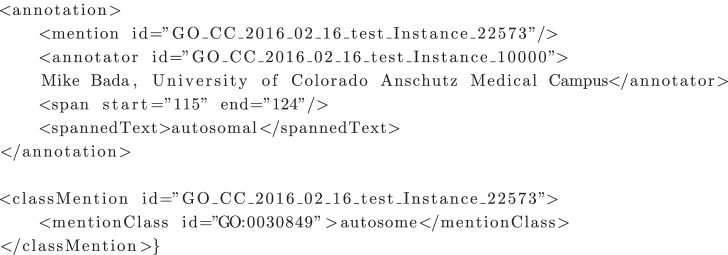


Here, the word *“autosomal”* with a character span of 115 - 124 is tagged to GO term “GO:0030849”. In order to obtain annotations per word, we utilize a sentence segmentation library called SpaCy (https://spacy.io/). First, the segmenter splits the text into sentences by accounting for sentence end marks (such as periods, exclamation, question marks, etc.) and then uses a tokenizer to split the sentences into individual words (or tokens) by accounting for word boundaries (such as space, hyphen, tab, etc.). For example, the above sentence is split into individual tokens as follows:

**Sentence:** *“We observed a severe autosomal recessive movement disorder in mice used within our laboratory.”*

**Tokens:** *[‘We’, ‘observed’, ‘a’, ‘severe’, ‘autosomal’, ‘recessive’, ‘movement’, ‘disorder’, ‘in’, ‘mice’, ‘used’, ‘within’, ‘our’, ‘laboratory’, ‘.’ ]*

**Annotation:** *{‘start’: 115, ‘end’: 124, ‘spanned_text’: ‘autosomal’, ‘go_term’: ‘GO:0030849’ }*

#### IOB Tagging

The deep learning models need to know if each individual word/token corresponds to a GO term. Each extracted word/token is mapped to a GO term or an *out-of-concept* annotation. Here we use the range specified in the xml to map the token to one of three tags: 1) ‘GO’ to indicate an annotation, ‘O’ for a non-annotation (out-of-concept), and ‘EOS’ to indicate the end of sentence. For example, the following sentence would be tagged as below:

**Sentence:** *“We observed a severe autosomal recessive movement disorder in mice used within our laboratory.”*

**Tokens:** *[ ‘We’, ‘observed’, ‘a’, ‘severe’, ‘autosomal’, ‘recessive’, ‘movement’, ‘disorder’, ‘in’, ‘mice’, ‘used’, ‘within’, ‘our’, ‘laboratory’, ‘.’ ]*

**IOB Tags:** [‘O’, ‘O’, ‘O’, ‘O’, ‘GO:0030849’, ‘O’, ‘O’, ‘O’, ‘O’, ‘O’, ‘O’, ‘O’, ‘O’, ‘O’, ‘EOS’]

The above example shows a simple case where a single word is annotated to a GO concept. In other cases, a sequence of words/tokens is annotated to a GO term. We utilize the IOB (Inside, Outside, Beginning) [30] standard for annotating multi-span tokens to account for such annotations. The IOB format uses three prefixes to tag tokens in a sentence: 1) ‘B-GO’ is used to specify the beginning of the annotation, 2) ‘I-GO’ is used to map the tokens following the beginning of annotation till the end, and 3) ‘O’ is used to map tokens that don’t correspond to a GO term. The following sentence shows an example of IOB formatting:

**Sentence:** *“The phosphatidylserine receptor primarily functions in apoptotic cell clearance.”*

**Annotation:** *{‘start’: 1862, ‘end’: 1886, ‘spanned_text’: ‘apoptotic cell clearance’, ‘go_term’: ‘GO:0043277’}]*

**Tokens:** *[ ‘The’, ‘phosphatidylserine’, ‘receptor’, ‘primarily’, ‘functions’, ‘in’, ‘apoptotic’, ‘cell’, ‘clearance’, ‘.’ ]*

**IOB Tags:** [ ‘O’, ‘O’, ‘O’, ‘O’, ‘O’, ‘O’, ‘B-GO:0043277’, ‘I-GO:0043277’, ‘I-GO:0043277’, ‘EOS’ ]

In the above example, the phrase *“apoptotic cell clearance”* is annotated to ‘GO:0043277’. We tag the token ‘apoptotic’ with ‘B-GO:0043277’ indicating the beginning of the annotation. The tokens ‘cell’ and ‘clearance’ are tagged with ‘I-GO:0043277’ indicating the continuation of the annotation. ‘O’ is used to map the rest of the tokens which do not correspond to any annotations and ‘EOS’ is used to map *‘.’* signifying the end of the sentence.

#### Annotation formats

Sentences in the CRAFT corpus are annotated following a set of annotation formats and guidelines as detailed in https://github.com/UCDenver-ccp/CRAFT/tree/master/concept-annotation. Below, we describe how sentences that contain annotations in different formats are represented in the IOB format.

- **No annotations**: Some sentences in an article might not contain any annotations. In this case, all tokens are represented by ‘O’ tags except the ending character which is represented by ‘EOS’ tag.
- **Disjoint annotations:** A sentence might contain one or more annotations that don’t overlap in terms of annotation span. In this case, all tokens not corresponding to an annotation are tagged with ‘O’ tags. The end of sentence character is represented by ‘EOS’ tag. Tokens that mark the the beginning of an annotation are marked with a ‘B-GO:term’ followed by ‘I-GO:term’ to represent subsequent tokens corresponding to an annotated phrase.
- **Overlapping annotations:** A sentence might contain a phrase (sequence of words/tokens) that is annotated to a GO concept, and a word or a sub-phrase within the original phrase that is annotated to a different GO concept. In these instances, we make *n* copies of the sentence where *n* is the number of different annotations. Each copy contains a modified sentence that represents the text needed to convey one of the annotations. If a sentence contains a case of overlapping annotations and other disjoint annotations (non-overlapping annotations), we create sentences that capture the different variations of the overlapping annotations while keeping the disjoint annotations common.
- **Multiple overlapping annotations**: Sentences can also have more than one phrase with sub-annotations. In such a case, where there exist *m* phrases with $n_1, n_2, \cdots , n_m$ overlapping subphrases, there will $n_1 \times n_2 \times ... \times n_m$ copies with all possible combinations of sub-phrase mappings.

Below we present examples of each of these annotation types and describe how they are processed for training the models:

- **No annotations**:
  **Sentence**: *“Rescue of Progeria in Trichothiodystrophy by Homozygous Lethal Xpd Alleles”*
  **Annotations**: {None}
  **IOB Tags**: [ ‘O’, ‘O’, ‘O’, ‘O’, ‘O’, ‘O’, ‘O’, ‘O’, ‘O’, ‘O’, ‘EOS’ ]
- **Disjoint annotations:**
  **Sentence**: *“A cell progressing from anaphase to cytokinesis (pink arrowheads).”*
  **Annotations**: {*‘anaphase’* — GO:0051322; ‘cytokinesis’ — GO:0000910}
  **IOB Tags**: [ ‘O’, ‘O’, ‘O’, ‘O’, ‘B-GO:0051322’, ‘O’, ‘B-GO:0000910’, ‘O’, ‘O’, ‘O’, ‘O’, ‘EOS’ ]
- **Overlapping annotations:**
  **Sentence**: *“Having excluded a direct role in ****vesicle formation ****and membrane fusion, annexin A7 might act by its property as Ca2+-binding protein”*
  **Annotations**: {*‘vesicle’* — GO:0031982; ‘vesicle formation’ — GO:0006900}
  The above example is represented as two sentences with each sentence representing one of the two annotations.
  **Sentence 1**: *“Having excluded a direct role in ****vesicle ****and membrane fusion, annexin A7 might act by its property as Ca2+-binding protein”*
  **Annotations**: {*‘vesicle’* — GO:0031982}
  **IOB Tags**: [ ‘O’, ‘O’, ‘O’, ‘O’,‘O’,‘O’, ‘B-GO:0031982’ ‘O’, ‘O’, ‘O’, ‘O’, ‘O’, ‘O’, ‘O’, ‘O’, ‘O’, ‘O’, ‘O’, ‘O’, ‘O’, ‘EOS’ ]
  **Sentence 2**: *“Having excluded a direct role in ****vesicle formation ****and membrane fusion, annexin A7 might act by its property as Ca2+-binding protein”*
  **Annotations**: {*‘vesicle formation’* – GO:0006900}
  **IOB Tags**: [ ‘O’, ‘O’, ‘O’, ‘O’,‘O’,‘O’, ‘B-GO:0006900’, ‘I-GO:0006900’, ‘O’, ‘O’, ‘O’, ‘O’, ‘O’, ‘O’, ‘O’, ‘O’, ‘O’, ‘O’, ‘O’, ‘O’, ‘EOS’ ]
- **Multiple overlapping annotations**:
  **Sentence**: *“Having excluded a direct role in ****vesicle formation ****and ****membrane fusion****, annexin A7 might act by its property as Ca2+-binding protein.”*
  **Annotations**: {*‘vesicle’* — GO:0031982; *‘vesicle formation’* — GO:0006900; *‘membrane’* — GO:0016020; *’membrane fusion’* — GO:0061025}
  In this example, we have two instances of overlapping annotations with two sub-phrase annotations each. This sentence would be transformed to four sentences that each represents a unique combination of annotations.
  **Sentence 1**: *“Having excluded a direct role in ****vesicle ****and ****membrane****, annexin A7 might act by its property as Ca2+-binding protein.”*
  **Annotations**: {*‘vesicle’* — GO:0031982; *‘membrane’* — GO:0016020}
  **IOB Tags**: [ ‘O’, ‘O’, ‘O’, ‘O’,‘O’,‘O’, ‘B-GO:0031982’ ‘O’, ‘B-GO:0016020’,‘O’, ‘O’, ‘O’, ‘O’, ‘O’, ‘O’, ‘O’, ‘O’, ‘O’, ‘O’, ‘O’, ‘EOS’ ]
  **Sentence 2**: *“Having excluded a direct role in ****vesicle formation ****and ****membrane****, annexin A7 might act by its property as Ca2+-binding protein.”*
  **Annotations**: {*‘vesicle formation’* — GO:0006900; *‘membrane’* — GO:0016020}
  **IOB Tags:**[ ‘O’, ‘O’, ‘O’, ‘O’,‘O’,‘O’, ‘B-GO:0006900’, ‘I-GO:0006900’, ‘O’,‘B-GO:0016020’, ‘O’, ‘O’, ‘O’, ‘O’, ‘O’, ‘O’, ‘O’, ‘O’, ‘O’,‘O’, ‘EOS’ ]
  **Sentence 3**: *“Having excluded a direct role in ****vesicle ****and ****membrane fusion****, annexin A7 might act by its property as Ca2+-binding protein.”*
  **Annotations**: {*‘vesicle’* — GO:0031982; *’membrane fusion’* — GO:0061025}
  **IOB Tags:**[ ‘O’, ‘O’, ‘O’, ‘O’, ‘O’, ‘O’, ‘B-GO:0031982’, ‘O’, ‘B-GO:0016025’, ‘I-GO:0016025’, ‘O’, ‘O’, ‘O’, ‘O’, ‘O’, ‘O’, ‘O’, ‘O’, ‘O’, ‘O’, ‘EOS’ ]
  **Sentence 4**: *“Having excluded a direct role in ****vesicle formation ****and ****membrane fusion****, annexin A7 might act by its property as Ca2+-binding protein.”*
  **Annotations**: {*‘vesicle formation’* — GO:0006900; *‘membrane fusion’* — GO:0061025}
  **IOB Tags**: [ ‘O’, ‘O’, ‘O’, ‘O’, ‘O’, ‘O’, ‘B-GO:0006900’, ‘I-GO:0006900’, ‘B-GO:0016025’,‘I-GO:0016025’, ‘O’, ‘O’, ‘O’, ‘O’, ‘O’, ‘O’, ‘O’, ‘O’, ‘O’, ‘O’, ‘EOS’ ]
- **Discontinuous annotations**:
  **Sentence**: *“Because the F7 is the most severely affected allele, it is possible that the difference between the heart and kidney levels is due to a developmental delay in ****v****/p ****formation****.”*
  **Annotations**: *“v formation”* — GO:0097084
  Here we see *“v formation”* is annotated to GO:0097084, whereas *“/p”* is not. In such a case we represent the sentence by removing the tokens/words which were not annotated (*“/p”*). This is done to represent the continuous span of the phrase to GO term mapping.
  **Transformed Sentence**: *“Because the F7 is the most severely affected allele, it is possible that the difference between the heart and kidney levels is due to a developmental delay in ****v formation****.”*
  **IOB tags**: [ ‘O’, ‘O’, ‘O’, ‘O’, ‘O’, ‘O’, ‘O’, ‘O’, ‘O’, ‘O’, ‘O’, ‘O’, ‘O’, ‘O’, ‘O’, ‘O’, ‘O’, ‘O’, ‘O’, ‘O’, ‘O’, ‘O’, ‘O’, ‘O’, ‘O’, ‘O’, ‘O’, ‘O’, ‘O’, ‘B-GO:0097084’, ‘I-GO:0097084’, ‘EOS’ ]

We acknowledge the representation of discontinuous annotations is not ideal. However given that the majority of annotations in CRAFT are continuous, we prioritized the data to follow the same pattern. Some sentences might have a combination of disjoint, overlapping and/or discontinuous annotations. These sentences are broken down to smaller cases with precedence in the order of - overlapping, discontinuous, and disjoint annotations. If there are overlapping annotations, they are treated first i.e., multiple copies of the sentence are created and mapped for their annotations. Then for each copy, the discontinuous annotations are handled while keeping the disjoint annotations common between the representations.

While creating multiple copies of the sentences can lead to over-sampling of such cases, the overall number of such sentences were very low in comparison to the total number of sentences present in the training data. 

#### POS tagging and token encoding

Following the tokenization and IOB tagging, we enrich training data with parts-of-speech (POS) information and a compressed character representation. POS tagging looks at the contextual information of the word based on the words surrounding it in a sentence or a phrase. Here we used the SpaCy POS tagger to evaluate and tag the tokens of sentences with 15 parts of speech tags — adjective, adposition (such as - in, to, during), adverb, auxiliary (such as - is, has done, will do, should do), conjunction, coordinating conjunction, determiner, interjection, noun, numeral, particle, pronoun, proper noun, punctuation, subordinating conjunction, symbol, verb, other (not annotated to any of the others), space.

While the POS tagging looks at the word level representation of the context, we also represent character level nuances of a token using character encodings. These encodings represent upper-case and lower-case characters with ‘C’ and ‘c’ respectively. Numbers are represented using an ‘N’ and punctuation (such as commas, periods, and dashes) are retained in the encoding. Character encodings enable a succinct representation of a token’s unique characters which can indicate named entities and aid in the model’s learning.

Here we show an example of a sentence tagged with POS and character representations.

**Sentence**: *“Smith-Lemli-Opitz syndrome (SLOS, MIM 270400), a relative common dysmorphology disorder, is caused by mutations in DHCR7 [2-5], which encodes for 7-dehydrocholesterol *$\triangledown$*7-reductase and catalyzes a final step of cholesterol biosynthesis.”*

**Character Representation**: [‘Ccc-Ccc-Ccc’, ‘ccc’, ‘(’, ‘CCC’, ‘,’, ‘CCC’, ‘N’, ‘)’, ‘,’, ‘c’, ‘ccc’, ‘ccc’, ‘ccc’, ‘ccc’, ‘,’, ‘cc’, ‘ccc’, ‘cc’, ‘ccc’, ‘cc’, ‘CCCN’, ‘[’, ‘N-N’, ‘]’, ‘,’, ‘ccc’, ‘ccc’, ‘ccc’, ‘N-ccc’, ‘U’, ‘ccc’, ‘ccc’, ‘c’, ‘ccc’, ‘ccc’, ‘cc’, ‘ccc’, ‘ccc’, ‘.’]

**Parts-of-Speech**: [‘NNP’, ‘NN’, ‘-LRB-’, ‘NNP’, ‘,’, ‘NNP’, ‘CD’, ‘,’, ‘,’, ‘DT’, ‘JJ’, ‘JJ’, ‘NN’, ‘NN’, ‘,’, ‘VBZ’, ‘VBN’, ‘IN’, ‘NNS’, ‘IN’, ‘NNP’, ‘XX’, ‘CD’, ‘,’, ‘,’, ‘WDT’, ‘VBZ’, ‘IN’, ‘NN’, ‘NN’, ‘CC’, ‘VBZ’, ‘DT’, ‘JJ’, ‘NN’, ‘IN’, ‘NN’, ‘NN’, ‘.’]

#### BioThesaurus encoding

In addition to POS and token encoding, which capture sentence and token level context present in the data, we also include information from existing large scale knowledge bases. The first data source we use is BioThesaurus [31], which is a database of protein and gene names mapped to the UniProt Knowledge base. The database contains over 2.8 million names/tokens from separate data sources and is well regarded as a comprehensive thesaurus mapping words to their molecular/biological entities. We query BioThesaurus for each of the tokens extracted from the articles. First, we map if a token is present (1) or absent (0) in the database. If a token is present, we map if it identifies as a protein name, biomedical terms, chemical terms, and/or macromolecule. Sometimes, a token can be identified to multiple categories. In the following example we show the mapping of a token as queried from the BioThesaurus:

**Sentence**: *“Hematopoiesis is precisely orchestrated by lineage-specific DNA-binding proteins that regulate transcription in concert with coactivators and corepressors.”*

**Tokens**:       [ ‘Hematopoiesis’, ‘is’, ‘precisely’, ‘orchestrated’, ‘by’, ‘lineage-specific’, ‘DNA-binding’, ‘proteins’, ‘that’, ‘regulate’, ‘transcription’, ‘in’, ‘concert’, ‘with’, ‘coactivators’, ‘and’, ‘corepressors’, ‘.’]

**Protein**:       [ 0, 1, 0, 0, 1, 0, 1, 1, 0, 0, 1, 1, 0, 0, 0, 1, 0, 0]

**Biomedical**:    [ 0, 0, 0, 0, 0, 0, 0, 0, 0, 0, 1, 0, 0, 0, 0, 0, 0, 0]

**Chemical**:       [ 0, 0, 0, 0, 0, 0, 0, 0, 0, 0, 0, 0, 0, 0, 0, 0, 0, 0]

**Macromolecule**: [ 0, 0, 0, 0, 0, 0, 0, 0, 0, 0, 0, 0, 0, 0, 0, 0, 0, 0]

#### Unified Medical Language System (UMLS) Encoding

Continuing with the information augumentation, we also query the UMLS [32] database for tokens extracted from the articles. UMLS is another comprehensive database of over 2 million names representing medical and bio-medical terms aggregrated from several databases such as NCBI taxonomy, Gene Ontology, the Medical Subject Headings (MeSH), OMIM, ICD-10-CM, SNOMED CT, and the Digital Anatomist Symbolic Knowledge Base.

Here we query the metathesaurus component of the database for the extracted tokens. Words/tokens associated with a UMLS term are encoded as 1 or 0 otherwise. If a phrase (sequence of tokens) is found in UMLS, all tokens from the phrase are encoded as 1. Below we show an example of the mapping:

**Sentence:** *“Hematopoiesis is precisely orchestrated by lineage-specific DNA-binding proteins that regulate transcription in concert with coactivators and corepressors.”*

**Tokens:** [ ‘Hematopoiesis’, ‘is’, ‘precisely’, ‘orchestrated’, ‘by’, ‘lineage-specific’, ‘DNA-binding’, ‘proteins’, ‘that’, ‘regulate’, ‘transcription’, ‘in’, ‘concert’, ‘with’, ‘coactivators’, ‘and’, ‘corepressors’, ‘.’]

**UMLS:** [ 1, 0, 0, 0, 0, 0, 0, 1, 0, 0, 1, 0, 0, 0, 0, 1, 0, 0]

Prior to preprocessing, CRAFT articles were divided using an 80-20 split to create training and testing data. Training and testing data were then processed into sentences, tokenized, translated different annotation formats, and encoding using BioThesaurus and UMLS. The training data is used for development of the deep learning models (described in the following section). Testing data is used to evaluate model performance.

### Deep learning architecture

**Fig. 1:**
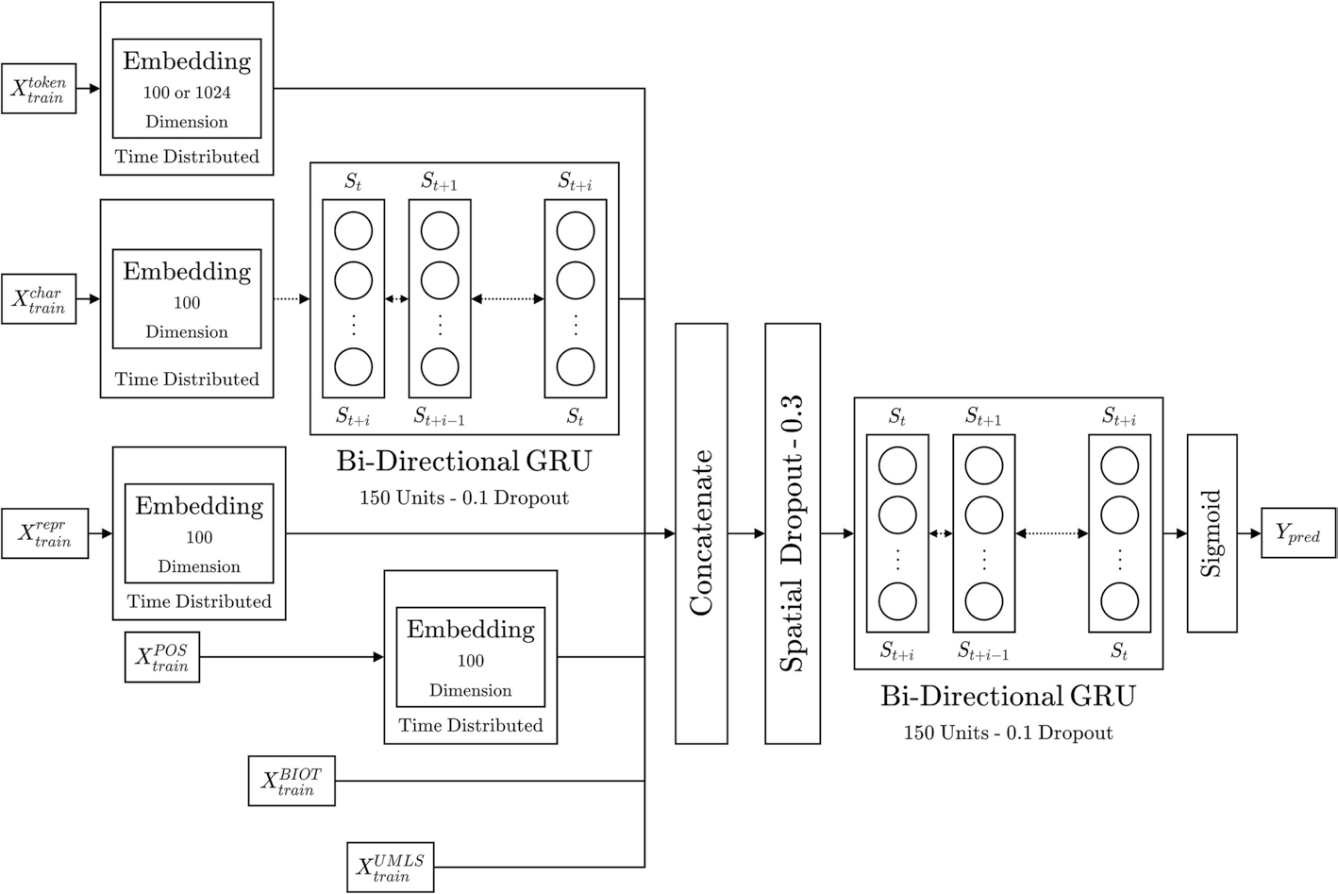
Architecture of a Gated Recurrent Unit (GRU) based model using multiple input pipelines

After all the preprocessing steps described above are complete, we develop multidimensional vectors for each sentence of the articles. Our deep learning architecture (Fig. 1) consists of three key components — 1) Input Pipelines; 2) Embedding/Latent Representations; and 3) Sequence Modeler. Below we describe each of the components:

#### Input pipelines

The recurrent neural architecture used in our approach requires fixed size inputs. Accordingly, we restrict each sentence to contain a maximum of 71 words/tokens. This is based on the third standard deviation of the distribution of frequency of words present in sentences. Sentences with lower number of words are padded with the token $\texttt {<PAD>}$ and ones with higher number of tokens are truncated to a length of 71. All corresponding input vectors are also adjusted accordingly to reflect the maximum sequence length representation of a sentence.

Each sentence and each token has six different components that are provided as input — 1) token ($X_{train}^{token}$), 2) character sequence ($X_{train}^{char}$), 3) token-character representation ($X_{train}^{repr}$), 4) parts-of-speech ($X_{train}^{POS}$), 5) BioThesaurus ($X_{train}^{BIOT}$), and 6) UMLS ($X_{train}^{UMLS}$).

The token ($X_{train}^{token}$) input, is a sequential tensor consisting of 71 tokens, where each token is represented with a high dimensional one hot encoded vector (for 34,164 unique words/tokens present within our corpus vocabulary). Apart from the $\texttt {<PAD>}$ token, we also use $\texttt {<UNK>}$ token to represent unknown tokens. This is done to generalize the model for words which were not available in the training data but can be present in testing dataset. Similarly, the character sequence ($X_{train}^{char}$) is also a sequential tensor consisting of character sequences present in a word/token. Here, we limit the maximum character length to 15 based on the third standard deviation of the character distribution. Tokens with longer sequences are truncated and tokens with shorter are padded with a $\texttt {<PAD>}$ character identifier. A single input sentence tensor for $X_{train}^{char}$ has a shape of (1,71,15), for 71 tokens and 15 characters.

Next we provide character representations ($X_{train}^{repr}$) and POS tags ($X_{train}^{POS}$). Both of these are based on words/tokens in sentences and are given as an input of 71 vectors. Biothesaurus encodings ($X_{train}^{BIOT}$) contain a four dimensional vector sequence where each token is one hot encoded for its association with protein, biomedical, chemical and macromolecule categories. UMLS encodings ($X_{train}^{UMLS}$) are also provided as an one hot encoded vector sequence, where 1 indicates a token’s presence and 0 indicates absence in UMLS.

#### Embedding/latent representations

Our architecture utilizes embeddings to provide a compressed latent space representation for very high dimensional input components. For example, the one hot vectorization of an individual word has a dimensionality of 34,166. In order to represent them succinctly and with contextual representation, we evaluated three different approaches for embeddings — 1) supervised embedding layer, 2) GloVe layer, 3) ELMo layer.

The supervised embedding is a bottleneck layer which learns to map the one hot encoded input into a smaller dimensional representation. The weights of this layer are learnt from the back propagation of losses based on the final output of the model. The resulting embedding learns the mapping of the IOB tags to the tokens of the sentences. The layer is used with token inputs ($X_{train}^{token}$), character sequences ($X_{train}^{char}$), character representation ($X_{train}^{repr}$), and POS tags ($X_{train}^{POS}$) each of which have very high dimensionality in their original vectors. We utilize 100 dimensional output representation for each of the aforementioned outputs, where weights are uniformly initialized at the start of the model training.

We also evaluate GloVe [33] and ELMo [34] pretrained embeddings for the $X_{train}^{token}$ input. Both are unsupervised approaches towards learning contextual representation of words from large scale corpora. GloVe uses word co-occurrence statistics to learn the embeddings. Pretrained data from cased Common Crawl with 840B tokens, 2.2M vocabulary, and 300 dimensional output embedding vector is used for this. In comparison, the embeddings in ELMo are learned via a bidirectional language model where the sequence of the words are also taken into account. We use the pretrained model on 1 Billion Word Benchmark, which consists of approximately 800M tokens of news crawl data and has an embedding of 1024 dimensional output embedding vector. While the embeddings are initialized from pretrained models, we allow for updates/retraining to the embedding models during the training of our larger model.

#### Sequence modeler

In order to model the input sequences, we utilize a deep bi-directional gated recurrent model (Bi-GRU). Bi-GRU was first proposed by Cho et. al. [35] as a more efficient approach than Long Short-Term Memory (LSTM) [36] while being able to tackle the vanishing gradient problem of vanilla Recurrent Neural Networks (RNN). The approach uses a gated mechanism to decide what information needs to be transmitted to the output of a single unit.

In our prior work [12, 37], we had evaluated multiple models based on RNN, LSTM, and GRU, and concluded that the GRU based architecture performed the best on CRAFT v2 annotation data. Building on that result, we employ the Bi-GRU as the base of our architecture in this work. As shown in Fig. 1, we utilize Bi-GRUs in two locations in the architecture, first to model the sequence of characters present in each token and a second main Bi-GRU model to concatenate input pipelines together. After the embedding of the characters, they are passed via the first Bi-GRU (consisting of 150 units) resulting in a sequence representation of the characters in a sentence. 10% dropout is used in this pipeline to regularize the output to prevent overfitting.

The character sequence representation is then concatenated with other embeddings, i.e. token (supervised/GloVe/ELMo), character representation, and parts of speech, and input tensors from Bio-Thesaurus and UMLS. This concatenated feature map representing each sentence is then passed to a spatial dropout, which removes 30% of the 1-D sequence features from the input to the main Bi-GRU. The main Bi-GRU processes the feature maps (with 10% dropout), and outputs to a single time-distributed dense layer of 1774 nodes (representing each of the output tags). A sigmoid activation is used in the last layer, where the final prediction is based on the highest probability value of the tags. There are 6 hidden layers and thus a total of 8 layers (including input and output layers) in our models. However, not all input pipelines go through all the layers. For instance, UMLS and PROT terms do not pass through the embedding layer.

**Fig. 2:**
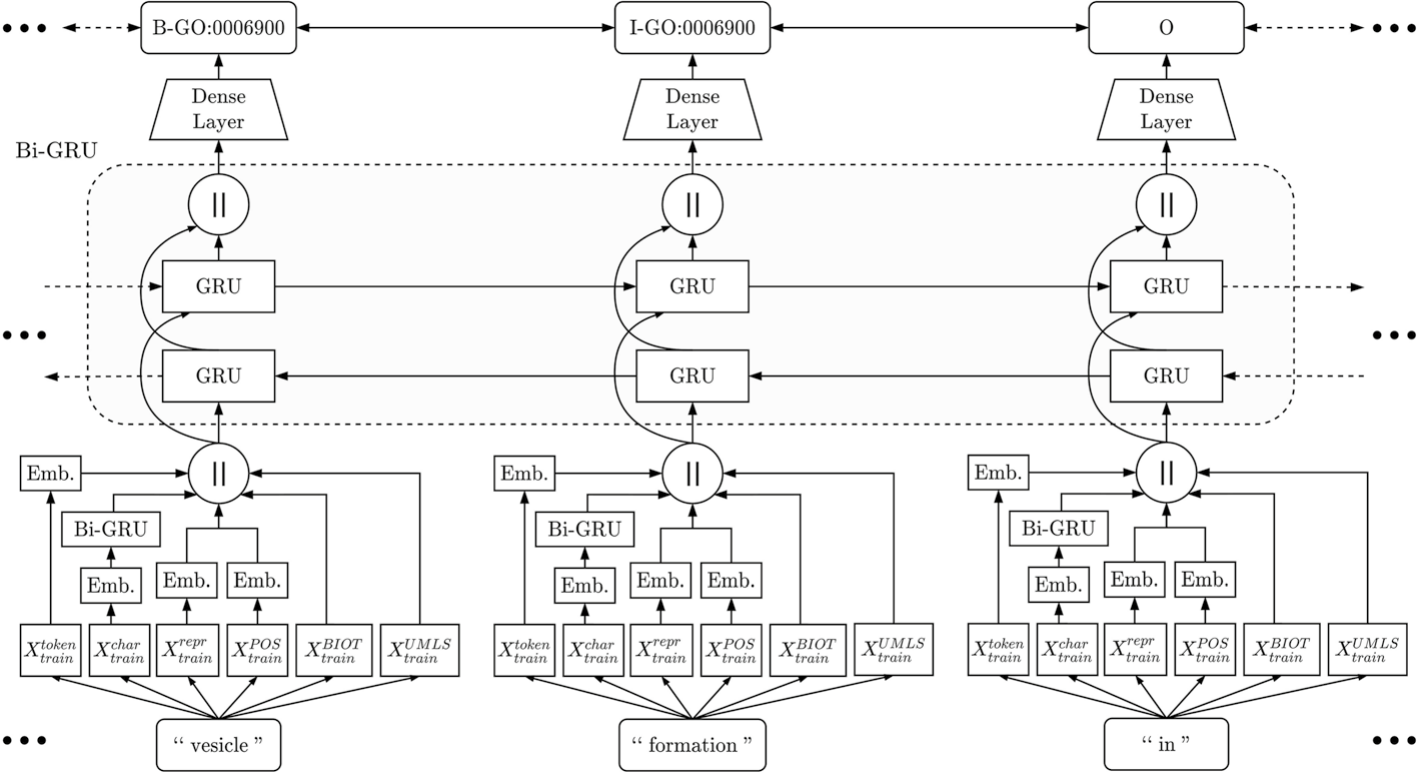
Workings of a GRU model with an example input sequence

Figure 2 shows a snapshot of the model architecture in the context of training and inference of a sample set of tokens. Here we show the training/inference on a sequence of tokens “vesicle”, “formation”, and “in” (which are parts of a sentence) as it is evaluated by the network. Each token is preprocessed to obtain the representative tensors – $X_{train}^{token}$, $X_{train}^{char}$, $X_{train}^{repr}$, $X_{train}^{POS}$, $X_{train}^{BIOT}$, and $X_{train}^{UMLS}$. $X_{train}^{token}$, $X_{train}^{char}$, $X_{train}^{repr}$, and $X_{train}^{POS}$ are passed through embedding layers, where the embedding of $X_{train}^{token}$ can be a complete pretrained architecture such as GloVe or ELMo. The embedding of $X_{train}^{char}$ is also passed via a Bi-directional GRU (Bi-GRU) layer. All of the resulting values are concatenated to be processed via the main Bi-GRU layer. Here we show each direction of the GRU layers as the process the input sequence. The first layer processes the sequence in its left to right ordering, i.e. “vesicle”, “formation”, and “in”, whereas the second layer processes the reverse, i.e. “in”, “formation”, and “vesicle”. The bi-directionality allows the architecture to learn the preceding and succeeding sequence patterns within the sequence tokens in a sentence. The states of both the GRU layers are then concatenated to provided to the final dense layer, which is the sigmoid classifier of the architecture, and predicts the associated IOB tags for the input tokens. Here we select the tag with the highest probability for each of the tokens.

We evaluated the impact of including each pipeline and token embedding approach to create nine different models that differ in the inputs pipelines provided to them. We evaluated three embeddings (CRAFT, GloVe, ELMo) in conjunction with these models to result in a total of 27 experiments. Architecture hyper-parameters, which include — supervised embedding shape ({20, 50, 100, 150, 200}), dropout ({0, .1, .2, .3, .5, .7}), number of epochs ({50, 100, 200, 300}), and class weighting, were evaluated using a grid search approach. We used Adam [38] as our optimiser for all of the experiments with a default learning rate of 0.0001. Learning rate reduction on plateau of loss was used, which reduced the rate by a factor of 0.1 if the loss stayed constant for 4 epochs. A batch size of 16 was used in all of our experiments.

Bidirectional Encoder Representations from Transformers (BERT) [39] is a popular attention model developed by Google. BERT has rapidly become the state of the art in several applications, especially those involving text processing. Instead of looking a text sequence in one direction, BERT uses bidirectional training which allows it to build better representations and context of textual inputs. The classic version of BERT was pretrained on a large corpus of English data. SciBERT, a variant of BERT, is trained on a large multi-domain corpus of scientific literature to improve performance on prediction of scientific entities. We compared the best model from our experiments with both versions of BERT.

### Performance evaluation metrics

The performance of each experiment is evaluated using a modified F1 score. The model is tasked with predicting non-annotations (indicated by an ‘O’ tag) or annotations (indicated by a ‘GO’ tag). Since the majority of tags in the training corpus are non-annotations, the model predicts them with great accuracy. In order to avoid biasing the F1 score, we omit accurate predictions of ‘O’ tags from the calculation to report a relatively conservative F1 score.

F1 quantifies whether the model’s prediction matches the actual annotation exactly. However, ontology-based prediction systems need to be evaluated while accommodating partially accurate predictions. For example, a model might not retrieve the exact ontology concept as the gold standard but a related concept (sub-class or super-class) achieving partial accuracy. Semantic similarity metrics [40] designed to measure different degrees of similarity between ontology concepts can be leveraged to measure the similarity between the predicted concept and the actual annotation to quantify the partial prediction accuracy. Here, we use Jaccard similarity [40] that measures the ontological distance between two concepts to assess partial similarity.

## Results and discussion

The CRAFT v4.0.1 dataset contains 18689 annotations pertaining to 974 concepts from the three GO sub-ontologies across 97 articles. Table 1 provides further information of the coverage of GO terms in CRAFT.

**Table 1:** Coverage of GO ontology concepts and annotations in the CRAFT corpus

GO sub-ontology	Concepts in ontology	Total annotations in CRAFT	Unique occurrences in CRAFT
Biological Process (BP)	30490	18392	710
Cellular Component (CC)	4463	6976	241
Molecular Function (MF)	12257	464	5

Table 2 shows the performance scores for Models 1 through 9 ($M_1$ - $M_9$) which differ in the inputs provided to them. $M_1$ is built with only tokens and no other inputs. Gradually, we add characters, character representation, parts of speech, and other inputs in each subsequent model. Each model is tested with three embeddings (CRAFT, GloVe, and ELMo). F1 and Jaccard semantic similarity are used to evaluate the models.

**Table 2:** Performance comparison of nine models based on GRUs with different input pipelines. Models are evaluated using F1 and semantic similarity. Each model includes certain inputs (listed as column headers). When a particular input type is included, there is a $\checkmark$ in the corresponding cell

Model	Input Pipelines									Embeddings					
	$X^{token}_{test}$	$X^{char}_{test}$	$X^{repr}_{test}$	$X^{POS}_{test}$	$X^{BIOT}_{test}$				$X^{UMLS}_{test}$	CRAFT		GloVe		ELMo	
					Prot.	Biom.	Chem.	Macr.		F1	Sem.	F1	Sem.	F1	Sem.
$M_1$	$\checkmark$									0.78	0.79	0.82	0.83	0.81	0.81
$M_2$	$\checkmark$	$\checkmark$								0.79	0.80	0.82	0.83	0.82	0.83
$M_3$	$\checkmark$	$\checkmark$	$\checkmark$							0.80	0.81	0.82	0.83	0.81	0.81
$M_4$	$\checkmark$	$\checkmark$	$\checkmark$	$\checkmark$						0.79	0.80	0.82	0.83	0.82	0.83
$M_5$	$\checkmark$	$\checkmark$	$\checkmark$	$\checkmark$	$\checkmark$					0.81	0.82	0.81	0.82	0.84	0.84
$M_6$	$\checkmark$	$\checkmark$	$\checkmark$	$\checkmark$	$\checkmark$	$\checkmark$				0.79	0.80	0.82	0.83	0.83	0.83
$M_7$	$\checkmark$	$\checkmark$	$\checkmark$	$\checkmark$	$\checkmark$	$\checkmark$	$\checkmark$			0.81	0.82	0.82	0.84	0.84	0.84
$M_8$	$\checkmark$	$\checkmark$	$\checkmark$	$\checkmark$	$\checkmark$	$\checkmark$	$\checkmark$	$\checkmark$		0.80	0.81	0.82	0.83	0.83	0.84
$M_9$	$\checkmark$	$\checkmark$	$\checkmark$	$\checkmark$	$\checkmark$	$\checkmark$	$\checkmark$	$\checkmark$	$\checkmark$	0.80	0.81	0.82	0.83	0.84	0.84

The base model with only tokens as input results in a F1 score in the range of 0.78 (for the CRAFT embedding) to 0.81 (GloVe and ELMo) and a semantic similarity of 0.81 to 0.82. The higher semantic similarity indicates that there are instances where the model misses the exact annotation in the gold standard yet predicts a partially related concept. These instances are captured and accounted for in the semantic similarity metric via partial credit whereas they receive a score of 0 in the F1 calculation.

Adding character sequences ($M_2$) improves F1 and semantic similarity scores across almost all embeddings. Adding token-character representation ($M_3$) yields mixed results. We see an improvement in F1 and Semantic similarity for the CRAFT embedding. However, both scores stay unchanged with GloVe and decrease with ELMo. The inclusion of parts of speech ($M_4$) causes a decrease in scores with CRAFT and ELMo. Both scores remain unchanged with GloVe. Providing protein names from BioThesaurus ($M_5$) improves both scores for CRAFT and ELMo while we observe a decrease in Glove. Here, we observe the highest F1 (0.84) and semantic similarity (0.84) across all models tested so far. $M_6$ - $M_9$ yield comparable results but do not result in further improvements over $M_5$. In summary, our best model resulted in an F1 score and semantic similarity score of 0.84 with the ELMo embedding.

**Fig. 3:**
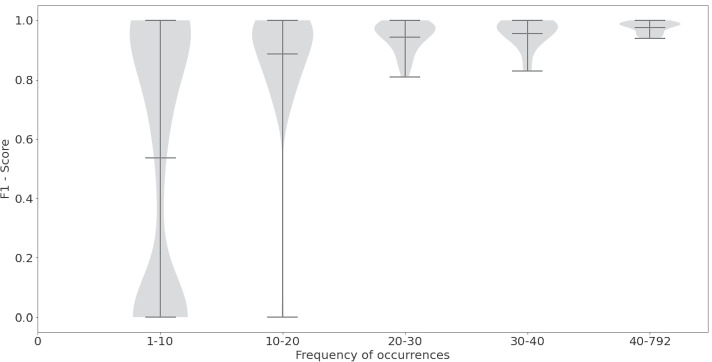
Distribution of F1 scores by occurrence frequency of GO terms in CRAFT

**Fig. 4:**
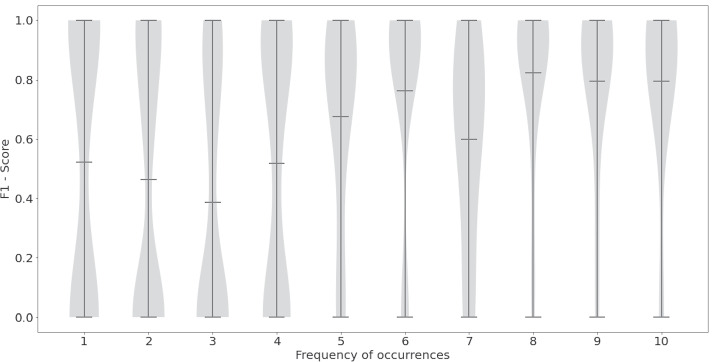
A closer look at the distribution of F1 scores for GO terms with 10 or less occurrences in CRAFT

We further analyzed our best model to gather insights into the model’s performance. First, we show the modified F1 score for the three GO sub-ontologies (Table 3). The model shows a similar performance for biological process and cellular component and registers a substantially higher score for molecular function. This might be because the total number of annotations from the molecular function sub-ontology in the CRAFT corpus is far lower than the other two ontologies (see Table 1). Table 4 shows the false positives, false negatives, true positives, and true negatives among our predictions broken down by the three GO sub-ontologies. Next, we explored if the occurrence frequency of a concept in the training corpus impacts the model’s prediction performance on that concept. Figure 3 breaks down the F1 score into five bins based on the GO terms’ frequency of occurrence in the corpus. We see that GO terms with a frequency of co-occurrence between 1-10 have substantial variability in their F1 scores. Most of the GO terms with 10-20 occurrences show F1 scores between 0.6 and 1. We see some outliers in this bin where the F1 scores are lower than 0.6. All bins with occurrences of 20 and higher show high F1 scores ($>0.8$) and low variability. This figure clearly shows that the model makes incorrect predictions for GO terms with low occurrences ($<20$) in the corpus. We did not observe evident differences in prediction performance when the 1-10 occurrence bin was further subdivided into smaller intervals (Fig. 4).

**Table 3:** Modified F1 scores from model M_9 broken down by GO sub-ontology

	GO_BP	GO_MF	GO_CC
M_9	0.82	0.96	0.85

**Table 4:** Confusion matrix for predictions by GO sub-ontology (Biological Process (GO_BP), Cellular Component (GO_CC), and Molecular Function (GO_MF). Note that this matrix does not include accurately predicted ‘O’ and ‘EOS’ tags since these instances are omitted during calculation of the modified F1 score. Results are from our best model M_9

True Class	Predicted Class					
		GO_BP	GO_MF	GO_CC	‘O’	‘EOS’
	GO_BP	3419	1	25	393	0
	GO_MF	0	96	1	2	0
	GO_CC	7	0	1288	116	0
	O	68	0	34	N/A	4
	EOS	0	0	0	0	N/A

We compared our best model with classic BERT as well as SciBERT (Table 5). We find that SciBERT performs better than BERT by 3 points in F1 and 2 points in semantic similarity. Our model improves SciBERT’s F1 by 4 points and semantic similarity by 2 points.

**Table 5:** Performance comparison between our best model and two variants of BERT

Model	F1	Semantic Similarity
BERT	0.77	0.80
SciBERT	0.80	0.82
$M_9$	0.84	0.84

The model predicted 83.61% of annotations in the test set accurately. 9.34% were prediction errors where the model mis-classified GO annotations as non-annotations (‘O’ tags). 1.72% were prediction errors where the model mis-classified ‘O’ tags as GO terms. Finally, in 5.32% of cases, the model predicted a different GO term than the GO term in the test corpus.

**Fig. 5:**
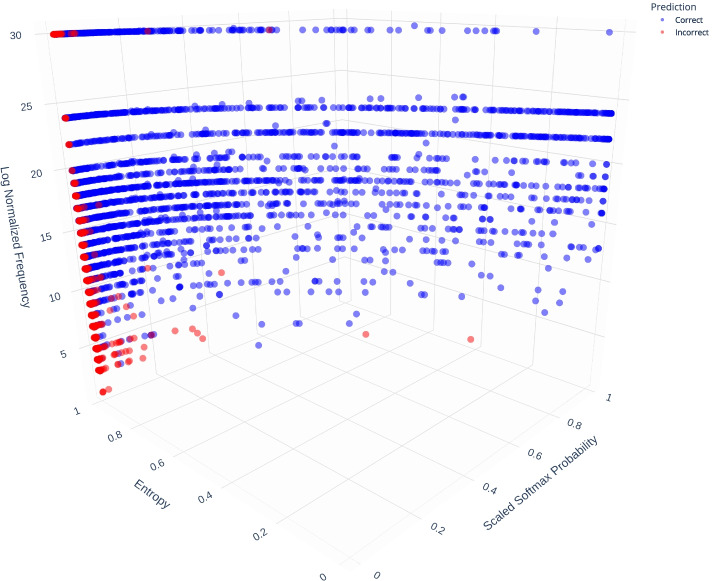
Distribution of incorrect and correct predictions with respect to entropy, probability, and frequency of occurrences

For each word in a sentence, the model outputs a tensor of sigmoid ($\frac{1}{(1 + \exp (-x_i))}$) activation outputs. These outputs are then converted to probabilities using a softmax function ($\frac{\exp (x_i)}{\sum _j \exp (x_j)}$). We can calculate the entropy (*H*(*X*)) over the tensor of probabilities to observe the level of “information” within the probabilities. For example, if there is uniformity in the probabilities for the predicted annotations, entropy is maximized, and vice versa. We visualized the interactions between entropy, predicted probability and the frequency of annotations, in Fig. 5. Here, the dots represent the predicted annotations (annotation with highest sigmoid activation) by the model. Incorrect predictions are shown in red and correct predictions in blue.

We observe that as the probability score increases (for the top prediction) and the entropy reduces (across prediction tensor), the model predictions are more accurate. The high probability of the top prediction indicates the model’s confidence and low entropy indicates that the model assigned low probabilities to the other potential predictions thereby offering a clear discrimination between the top prediction and the rest.

**Fig. 6:**
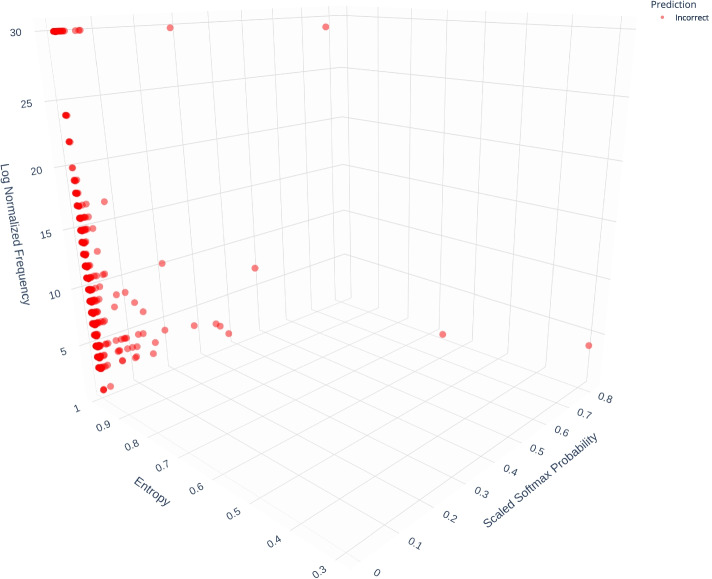
Distribution of incorrect predictions with respect to entropy, probability, and frequency of occurrences

In comparison, incorrect predictions (Fig. 6) are concentrated in a small area demarcated by low probability, high entropy, and low frequency. These incorrect predictions happen overwhelmingly at frequencies under 10 and at probability values lower than 0.1. The entropy values of the majority of these predictions is close to 1 indicating that the model assigned near uniform probabilities to the potential predictions. This combined with the low probability indicates that the model was not confident of any of the predictions it made.

We tested if there are differences in the entropy, frequency, and probability distributions between correct vs. incorrect predictions using two-sided independent T-tests. We found statistically significant differences at the Bonferroni-corrected threshold of $\alpha = 0.01$ between correct vs. incorrect predictions for entropy (*p* = 1.5e-221), frequency of occurrence (*p* = 2.9e-20), and probability of highest prediction (*p*=0.0).

We cannot use our results as a direct benchmark against other studies since the CRAFT corpus version used here might vary but we can remark on a general comparison. Lenz et al [29] developed a concept recognition system based on LSTMS which resulted in a 0.81 F1 (averaged across the three GO ontologies) as compared to our best model that results in a 0.84 F1 score. In a 2017 paper, OGER (OntoGene’s Entity Recognizer)’s method for concept recognition uses dictionary lookup and flexible matching reporting a F1 score of 0.70 [41]. A systematic evaluation [42] found that ConceptMapper [43] a dictionary-lookup system performed the best among other tools resulting in a F1 of 0.83 (using earlier versions of CRAFT).

## Future work

While the models presented here accurately predicted about 83% of annotations in the test set, there is substantial room for improvement in the remaining 17% where the model made prediction errors. One of the goals is for these models to make ontology-aware predictions. This means that in cases where the model fails to make an exactly accurate prediction, it should predict a closely related ontology concept (such as a parent or a super-class). We found that in cases where the model predicts a GO term that is different from the ground truth, the mean semantic similarity is a meagre 0.08 indicating that there is scant partial similarity between the incorrect predictions and the ground truth. Our future work will focus on moving incorrect predictions closer to the ground truth by creating ontology-aware models that take the ontology’s hierarchy into account.

## Data Availability

The data used for this study is publicly available at http://bionlp-corpora.sourceforge.net/CRAFT/. The code and models developed in this study are publicly available at https://github.com/prashanti/deeplearningNER.
